# Biomechanical Usability Evaluation of a Novel Detachable Push–Pull Device for Rehabilitation in Manual Wheelchair Users

**DOI:** 10.3390/life15071037

**Published:** 2025-06-30

**Authors:** Dongheon Kang, Seon-Deok Eun, Jiyoung Park

**Affiliations:** 1 Assistive Technology Research Team for Independent Living, Rehabilitation Research Institute, National Rehabilitation Center, Ministry of Health and Welfare, Seoul 01022, Republic of Korea; luxpooh@gmail.com; 2 Department of Safety and Health, Wonkwang University, Iksan 54538, Republic of Korea

**Keywords:** manual wheelchair, dual-propulsion, push–pull mechanism, biomechanics, electromyography

## Abstract

Manual wheelchair users are at high risk of upper limb overuse injuries due to repetitive propulsion mechanics. To address this, we developed a novel detachable push–pull dual-propulsion device that enables both forward and backward propulsion, aiming to reduce shoulder strain and promote balanced muscle engagement. This study presents a protocol to evaluate the device’s biomechanical impact and ergonomic effects, focusing on objective, quantitative analysis using a repeated-measures within-subject design. Thirty participants with spinal cord injury will perform standardized propulsion trials under two conditions: push and pull. Motion capture and surface electromyography (EMG) will assess upper limb kinematics and muscle activation. Each propulsion mode will be repeated over a 10-m track, and maximum voluntary contraction (MVC) data will be collected for EMG normalization. The protocol aims to provide objective evidence on the propulsion efficiency, muscle distribution, and ergonomic safety of the device. Findings will inform future assistive technology development and rehabilitation guidelines for manual wheelchair users.

## 1. Introduction

Manual wheelchair propulsion imposes significant physical demands on the upper limbs, frequently resulting in chronic shoulder pain, muscular imbalances, and overuse injuries, particularly among individuals with spinal cord injury (SCI) [1,2,3]. The repetitive nature of traditional forward propulsion exacerbates the risk of secondary musculoskeletal complications, which can adversely affect users’ quality of life and functional independence [4,5]. These cumulative risks highlight the pressing need for innovative propulsion solutions that can alleviate upper limb stress without compromising independent mobility.

Various alternative propulsion mechanisms have been explored to mitigate these issues and improve propulsion efficiency. Lever-based wheelchair propulsion, for instance, has demonstrated benefits such as altering muscle activation patterns, reducing peak muscular loads, and redistributing muscular demands [6]. le Rütte et al., (2022) showed that a push–pull lever mechanism significantly reduced peak propulsion forces and energy expenditure compared to conventional hand rim propulsion, highlighting its ergonomic advantages [7]. Furthermore, recent research on reverse propulsion has shown significant reductions in superior and posterior shoulder forces [8,9,10], highlighting the importance of propulsion direction on upper limb biomechanics and muscle activity [11] compared to traditional propulsion methods.

Building upon these findings, our research team has developed a novel detachable push–pull dual-propulsion device designed to integrate the biomechanical advantages of pushing and pulling methods, facilitating easy attachment to and detachment from conventional manual wheelchairs. This newly developed device is anticipated to enhance propulsion efficiency, reduce shoulder loading, and promote balanced muscle activation patterns compared to traditional propulsion methods. Moreover, its detachable design enables integration with standard wheelchairs, allowing for broader usability without structural modification. These anticipated benefits require objective verification through the biomechanical evaluation protocol presented in this study.

However, a comprehensive and rigorous usability assessment protocol is essential before implementing it more broadly. Current usability assessment protocols commonly integrate multimodal biomechanical measurements, including kinematic motion analysis, electromyography (EMG), and kinetic analysis. Bontrager et al., (2008), for example, utilized EMG to assess shoulder muscle loads, while Kukla et al., (2022) combined 3D motion analysis and EMG to evaluate the biomechanical impacts of innovative wheelchair propulsion mechanisms [6,12]. Recent studies have emphasized the importance of integrating objective performance-based metrics in assistive device evaluation, particularly through synchronized biomechanical measurements such as motion capture and EMG [13]. Despite such advances, few studies have employed rigorous, standardized protocols to examine dual-mode propulsion in realistic propulsion scenarios.

Thus, this study proposes a structured protocol for comprehensively assessing the usability of the novel detachable push–pull dual-propulsion device. This protocol integrates biomechanical analyses, including kinematic motion capture and electromyography (EMG), to rigorously evaluate the effects of propulsion methods on upper limb load, muscle activity, and propulsion efficiency. The protocol also aims to establish baseline biomechanical profiles that may inform both device refinement and clinical decision-making for safe and effective wheelchair propulsion. Focusing on biomechanical efficiency, this protocol aims to generate evidence-based insights that can inform future wheelchair design and clinical recommendations to enhance the health and functional mobility of manual wheelchair users. Ultimately, these findings could support evidence-based rehabilitation strategies and contribute to the design of future assistive technologies tailored to user-specific biomechanical needs.

## 2. Materials and Methods

### 2.1. Participants

A total of 30 adults diagnosed with SCI will be recruited for this study. The inclusion criteria will be as follows: participants will be adults between the ages of 20 and 60 years who will have been diagnosed with spinal cord injury (SCI). They must have used a manual wheelchair regularly for at least six months and will be required to have the ability to propel a wheelchair independently over a 10-m track. Additionally, participants must not have any acute or chronic impairments affecting their upper limbs and will need to provide written informed consent before participation.

The exclusion criteria will be defined as follows: individuals will be excluded if they have neurological conditions other than SCI, current upper limb fractures, clinically diagnosed shoulder pathology, or other musculoskeletal disorders that could interfere with propulsion. Further exclusion criteria will include active shoulder pain, cognitive impairments that limit comprehension of study instructions, and prior participation in competitive wheelchair sports or structured upper limb training programs within the past six months. Any additional medical condition identified by the research team as potentially compromising participant safety or data integrity will also result in exclusion.

Before enrollment, all participants will receive detailed information about the study’s purpose, procedures, risks, and benefits, and written informed consent will be obtained.

### 2.2. Study Design

**Figure 1:**
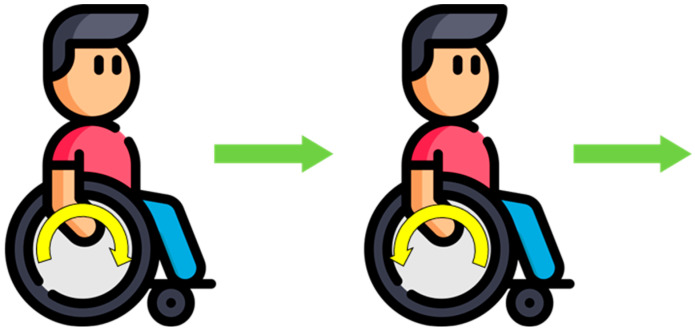
Illustration of the two experimental propulsion conditions. This visual was designed using resources from Flaticon.com.

This study will employ a repeated-measures within-subject design to evaluate the usability and biomechanical effects of a detachable push–pull dual-propulsion device developed for manual wheelchair users. Each participant will perform wheelchair propulsion tasks under two specific experimental conditions: (1) forward propulsion using the traditional handrim method (standard manual wheelchair), and (2) backward propulsion using the dual-propulsion device (pull mode). To enhance clarity, a schematic illustration (Figure 1) will be provided to visually depict the two experimental conditions: push propulsion using a handrim and pull propulsion using the dual-propulsion device. The arrows in the figure will indicate the direction of propulsion and the rotation of the wheels.

These two propulsion strategies will be selected to distinguish the mechanical and physiological differences between conventional pushing and the novel pulling movements using the developed device. This within-subject comparison will enable the evaluation of the dual-propulsion system in relation to standard propulsion, with a focus on biomechanics and usability.

While this study will adopt a within-subject repeated-measures design to compare two propulsion modes, we acknowledge the absence of a separate control group as a limitation. In future randomized controlled trials, we plan to include a conventional propulsion-only control group to improve external validity and control for potential confounding variables.

The order of propulsion conditions will be randomized for each participant to minimize potential learning effects, fatigue accumulation, and sequence bias. Prior to data collection, all participants will receive standardized instructions and practice sessions on the use of the propulsion device and experimental procedures to ensure sufficient familiarity.

All experiments will be conducted in a controlled laboratory setting. The primary outcome measures will include kinematic data obtained from 3D motion analysis and electromyographic (EMG) signals collected from upper limb muscles. Adequate rest intervals will be provided between conditions to prevent muscular fatigue and maintain the accuracy of the data.

**Figure 2:**
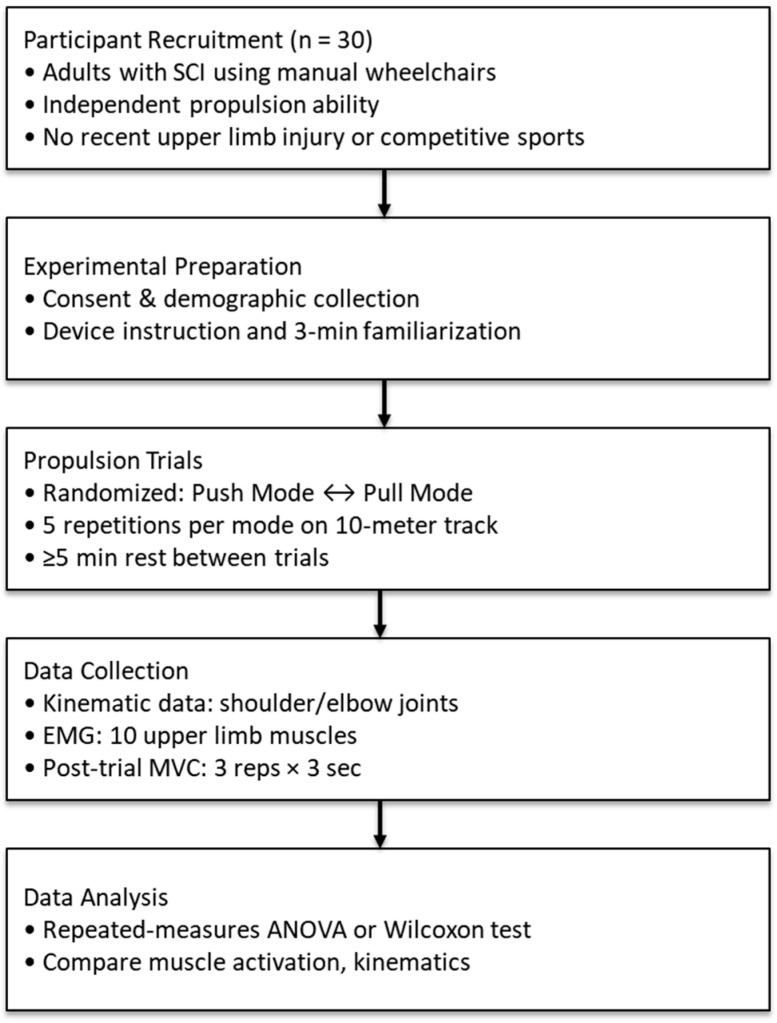
Study flowchart.

This study protocol was reviewed and approved by the Institutional Review Board of the National Rehabilitation Center (Approval No. NRC-2024-04-018). It was designed in accordance with the SPIRIT 2013 guidelines (Standard Protocol Items: Recommendations for Interventional Trials) [14]. A flowchart will illustrate the sequential steps of the experimental protocol (Figure 2), including participant recruitment, familiarization with the propulsion device, randomized push and pull trials along a 10-m track, and synchronized collection of kinematic and electromyographic (EMG) data. Maximum voluntary contraction (MVC) values will be recorded post-trial for EMG normalization.

The primary hypothesis of this study is that the pull-mode propulsion using the dual-propulsion device will result in more balanced upper limb muscle activation and reduced shoulder loading compared to standard push-mode propulsion.

### 2.3. Dual-Propulsion Device

The detachable push–pull propulsion device is engineered to be mounted on a standard manual wheelchair frame via a secure locking mechanism. It allows users to switch between traditional push propulsion and a novel pull-based mode using a detachable lever-arm structure designed for efficient biomechanical engagement. The system is compatible with standard active-type wheelchairs, enabling users to alternate propulsion strategies based on individual needs or task demands.

**Figure 3:**
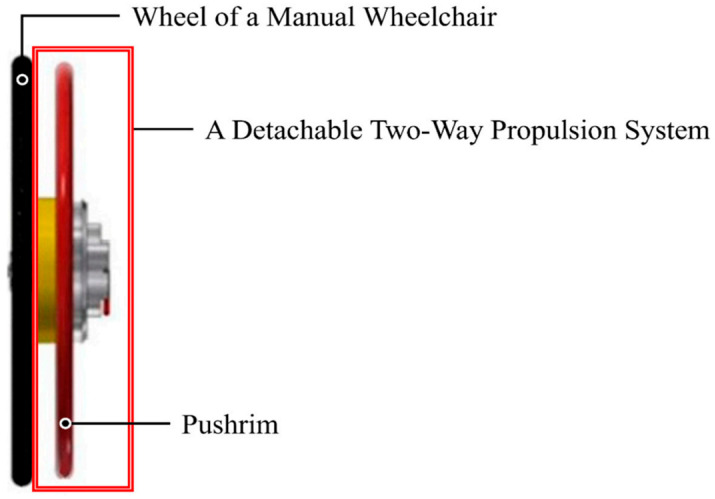
Side view of the key components in the detachable two-way propulsion system [15].

Figure 3 provides a side view of the key structural components of the detachable two-way propulsion system. The figure illustrates the pushrim, the detachable mechanical unit for bidirectional propulsion, and its integration with a standard manual wheelchair wheel. This figure will help visualize how the device is mounted and how the propulsion interface is layered over the existing wheel structure.

The dual-propulsion device used in this study is a detachable bidirectional propulsion mechanism that enables both forward (push) and backward (pull) propulsion for manual wheelchair users. This device was developed to reduce upper limb overuse by engaging different muscle groups depending on the direction of propulsion, thereby inducing biomechanical variation and offering ergonomic benefits to the user [15].

The system consists of a central drive module mounted to the wheelchair frame and a pair of rotating levers attached to each rear wheel. These levers are connected through a gear-based linkage system that transmits power to the wheels during both pushing and pulling actions. A mode-switching mechanism, operated via a lever or switch, allows the user to intuitively alternate propulsion directions.

**Figure 4:**
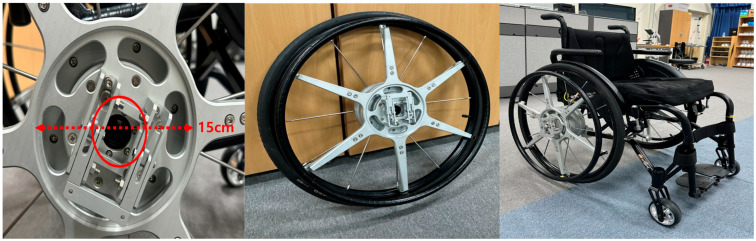
Prototype of the detachable push–pull dual-propulsion device mounted on a manual wheelchair [15]. The system comprises lever arms, gear mechanisms, and a central mounting module that is compatible with standard wheelchair frames. The red circle shows the quick-release axles as applied in the two-way propulsion system. Scale bar = 15 cm.

To ensure independent use and safety, the device includes a locking mechanism to secure the stationary levers and a quick-release system for easy attachment and detachment. This design permits installation without requiring structural modification to the existing wheelchair. The device is compatible with most active-type manual wheelchairs, and the prototype used in this study was based on the Nissin NA-430 model (Figure 4).

The handles and gear mechanism are optimized for user comfort and mechanical efficiency. The device’s total weight is approximately 3.6 kg, and its addition to the wheelchair does not significantly increase the overall width, thus maintaining maneuverability in daily activities. This study will validate the device’s biomechanical effects and propulsion efficiency using a structured experimental protocol.

The detachable push–pull dual-propulsion device includes a secure clamp-lock mechanism that attaches to standard wheelchair frames. Stability is ensured through a dual-locking gear system that prevents unintentional disengagement. Load-bearing components are constructed from lightweight aluminum alloy tested according to ISO 7176 [16] durability standards, and the lever mechanism features an anti-rebound damper to avoid sudden backward motions.

### 2.4. Experimental Protocol

This study will employ a repeated-measures within-subject design to evaluate the propulsion efficiency and biomechanical characteristics of a detachable push–pull dual-propulsion device developed for manual wheelchair users. The experiment will be conducted in a controlled laboratory setting at the National Rehabilitation Center, where each participant will perform two propulsion conditions: (1) forward (push) propulsion and (2) backward (pull) propulsion. Each condition will be repeated five times, resulting in ten trials of propulsion per participant. The order of the conditions will be randomized for each individual to minimize potential learning or fatigue effects.

The experimental session will consist of several sequential phases, including informed consent, familiarization, propulsion trials (push and pull), rest periods, and MVC measurements. A structured participant timeline will be followed to ensure procedural consistency across subjects, as outlined in Table 1. Each propulsion condition will be completed five times over a 10-m track, with adequate rest provided between trials. After the propulsion trials, maximum voluntary contraction (MVC) values will be collected for EMG normalization.

The experiment will take place along a 10-m straight indoor track. Before data collection, participants will receive a 3-minute familiarization session with the wheelchair equipped with the dual-propulsion device. During this time, participants will be instructed on how to operate the device and perform the experimental tasks. Adequate rest periods will be ensured throughout the protocol to minimize muscular fatigue. Upon completion of all propulsion tasks, MVC measurements will be obtained for each targeted muscle group, with each MVC performed three times for three seconds per trial.

Upper limb kinematic data and electromyographic (EMG) signals will be recorded during the propulsion tasks. Three-dimensional motion analysis will be conducted using the VICON system (Oxford, UK) with 10 fixed cameras. Reflective markers will be placed at anatomical landmarks to enable precise tracking of shoulder and elbow joint angles and movement trajectories. Surface EMG data will be collected using the Trigno Wireless EMG System (Delsys Inc., Natick, MA, USA), targeting 10 upper limb muscles on the dominant side. Electrodes will be positioned according to SENIAM guidelines, except where alternative validated placements are used. All data will be synchronized and processed using Visual 3D software (version 6.0, C-Motion, Germantown, MD, USA).

This experimental design aims to quantitatively assess differences in muscle activation, joint motion, and propulsion dynamics between forward and backward propulsion and to objectively evaluate the usability and biomechanical advantages of the proposed dual-propulsion device.

Table 1 outlines the sequential phases of the experimental session, including pre-test procedures (consent and demographics), device familiarization, propulsion trials using push and pull modes, designated rest intervals, and post-test MVC measurements for EMG normalization. Each propulsion condition was performed five times over a 10-m track, with synchronized kinematic and EMG recordings.

### 2.5. Outcome Measures

The outcome measures of this study are designed to evaluate the biomechanical differences in upper limb function during two propulsion modes: forward (push) and backward (pull). These include both kinematic and muscle activation variables.

Kinematic variables comprise joint angles, range of motion (ROM), and stroke trajectories, with a particular focus on the shoulder and elbow during the propulsion cycle.

Muscle activity will be evaluated using surface electromyography (sEMG), focusing on activation intensity and timing of the upper limb muscles involved in propulsion. Normalized iEMG and RMS values will serve as the primary metrics for inter-condition comparison. All EMG data are normalized to MVC, which is measured post-trial.

### 2.6. Procedures

Kinematic data will be collected using a VICON motion capture system (Oxford, UK) equipped with 10 fixed cameras. Reflective markers will be placed on anatomical landmarks of the shoulder, elbow, wrist, and hand to capture movement during each propulsion cycle. The recorded data will be processed with Visual 3D software (C-Motion, Germantown, MD, USA) to extract joint angles, ROM, and stroke trajectories. Sampling will be conducted at 100 Hz, and all signals will be low-pass filtered before analysis.

sEMG signals will be recorded using a Trigno Wireless EMG system (Delsys Inc., Natick, MA, USA) from ten upper limb muscles on the dominant side: anterior, middle, and posterior deltoid; biceps brachii; triceps brachii; pectoralis major; upper, middle, and lower trapezius; and latissimus dorsi. Surface electrodes will be positioned according to SENIAM guidelines, ensuring consistent and valid placement across participants. However, the SENIAM guidelines do not provide placement recommendations for the pectoralis major and latissimus dorsi muscles. For the pectoralis major, electrodes will be placed approximately one-third of the distance from the suprasternal notch to the anterior axillary line, following the method described by Król et al., (2007) [17]. For the latissimus dorsi, electrodes will be aligned parallel to the muscle fibers, approximately 4 cm below the inferior angle of the scapula, as validated in a previous study [18].

EMG signals will be sampled at 2000 Hz and processed through bandpass filtering (20–450 Hz), full-wave rectification, and smoothing using a root mean square (RMS) algorithm with a 100 ms moving window. Both integrated EMG (iEMG) and RMS values will be calculated to provide complementary insights: iEMG will reflect the cumulative muscle activity over time, while RMS will highlight instantaneous variations in muscle activation.

Maximum voluntary contractions (MVCs) will be measured post-trial and will be used to normalize all EMG signals for comparability. Although MVCs are commonly performed before experimental tasks, in this study, they will be conducted after the propulsion trials to prevent anticipatory muscle activation or learning effects that may influence natural propulsion patterns. Participants will be given a sufficient rest period after the trials to mitigate any fatigue effects before performing MVCs. For each muscle, MVC signals will be collected in three separate trials, each lasting 5 s, with 1-min rest intervals between trials to avoid fatigue. Participants will be seated in their manual wheelchairs, with their trunks supported and their arms positioned as needed for each targeted muscle. Verbal encouragement will be provided during each trial to help participants exert their maximum effort.

- For the deltoid (anterior, middle, posterior), participants will perform isometric shoulder flexion, abduction, and horizontal extension against resistance, respectively.
- For the biceps brachii and triceps brachii, MVCs will be obtained during elbow flexion and extension against manual resistance at a 90° elbow angle.
- For the pectoralis major, MVC will be performed via horizontal adduction against resistance with the arm abducted at 90°.
- For the trapezius (upper, middle, lower), shoulder shrug, scapular retraction, and scapular depression will be tested, respectively, while sitting.
- For the latissimus dorsi, isometric shoulder extension will be performed against resistance with the arm extended backward.

Temporal synchronization between motion and EMG data will be achieved using a TTL signal transmitted via the motion system’s analog trigger module, ensuring signal alignment across both systems. Each participant will complete propulsion trials in both modes, and three successful propulsion cycles will be recorded per condition for analysis.

### 2.7. Data Analysis

All collected data will be analyzed using SPSS Statistics software (version 26.0, IBM Corp., Armonk, NY, USA). Descriptive statistics (mean ± standard deviation) will be used to summarize participant characteristics and all outcome measures.

To assess the normality of the data distribution, the Shapiro–Wilk test will be conducted. Based on these results, either parametric or non-parametric tests will be used. For normally distributed data, repeated-measures analysis of variance (ANOVA) will be conducted to compare biomechanical parameters (e.g., joint angles, ROM, iEMG, RMS values) between the two propulsion modes (push vs. pull). Mauchly’s test will be performed to assess the assumption of sphericity, and if violated, the Greenhouse–Geisser correction will be applied. Signals will be sampled at 2000 Hz.

For non-normally distributed variables, the Wilcoxon signed-rank test will be applied. All statistical analyses will adopt a two-tailed significance level of *p* < 0.05. To assess the magnitude of differences between conditions, effect sizes will be reported (Cohen’s d or partial eta squared, as appropriate).

Additionally, kinematic variables such as shoulder flexion and extension angles, as well as stroke cadence, will be calculated over complete propulsion cycles. EMG signals will be band-pass filtered (20–450 Hz), full-wave rectified, and smoothed using the root mean square (RMS) method with a 100-ms moving window. Integrated EMG (iEMG) data will also be computed to capture the total muscle activation over each propulsion cycle. EMG data will be normalized to maximum voluntary contraction (%MVC). Comparisons of muscle activity patterns will be made across propulsion modes to evaluate biomechanical efficiency, with a focus on reduced activation in shoulder prime movers during the pull phase.

## 3. Discussion

This protocol outlines a structured approach to evaluating the usability and biomechanical performance of a novel detachable dual-propulsion device for manual wheelchair users. It incorporates synchronized 3D motion capture and surface electromyography (EMG) to analyze upper limb movement and muscle activity. By employing a within-subjects design, the study is structured to enable direct comparisons between push and pull propulsion conditions under controlled laboratory settings. While this approach facilitates individual-level comparisons, appropriate statistical methods (parametric or non-parametric) will be applied based on the distribution of collected data [19,20].

Previous studies have reported that repetitive pushrim propulsion generates cumulative strain on upper limb joints—especially the shoulder complex—resulting in high rates of rotator cuff injuries, shoulder impingement, and chronic overuse conditions among long-term manual wheelchair users [1,3,21]. To address these concerns, alternative propulsion systems such as lever-driven mechanisms and reverse propulsion have been explored to help redistribute mechanical load and reduce the risk of upper limb injury. For example, lever-based systems have demonstrated effectiveness in reducing peak handrim forces and joint loading by offering a mechanical advantage through longer moment arms and more continuous force application during propulsion [6,22].

Recent studies on push–pull lever propulsion systems have shown that combining forward pushing and backward pulling enhances propulsion efficiency while distributing muscular demand more evenly between anterior and posterior upper limb muscles [7]. For instance, le Rütte et al. reported that a centrally mounted push–pull lever system significantly reduced peak propulsion forces and energy expenditure compared to traditional handrim propulsion, while also promoting smoother force application and a lower perception of strain [7]. These findings provide empirical support for the rationale behind our device design, which incorporates push–pull mechanics in a detachable form that can be retrofitted to existing manual wheelchairs.

Beyond the magnitude of force, the direction and pattern of muscle recruitment during propulsion play a key role in injury prevention. Reverse propulsion studies have demonstrated favorable changes in muscle activation patterns—specifically, reduced strain on frequently overused anterior muscles such as the deltoid and pectoralis major and increased activation of posterior muscles like the trapezius and latissimus dorsi [11,23]. In another study, Jahanian et al. examined a geared wheelchair wheel design and found that it reduced shoulder joint forces during propulsion under high-resistance conditions, such as ramp ascent [24]. The dual-propulsion system proposed in this protocol incorporates both forward and backward propulsion, which is hypothesized to shift the muscular workload across phases of movement dynamically. This redistribution may help lower the cumulative load on any single muscle group and improve long-term shoulder health among users.

However, the advantages of pull propulsion are not consistent across all device designs. For example, Rocha et al. reported that although novel geared wheelchair wheels reduced the number of propulsion strokes required, novice users exhibited higher energy expenditure and perceived exertion compared to those using traditional handrim wheels [25]. These mixed findings emphasize the need for device-specific biomechanical evaluations. In contrast to rowing-style systems, our device employs a direct push–pull lever mechanism that minimizes frictional losses and supports natural upper limb motion throughout both propulsion phases. Furthermore, our protocol includes a structured orientation and practice period designed to mitigate novelty-related effects and enable participants to become biomechanically accustomed to the dual-propulsion system.

From an injury prevention perspective, distributing propulsion effort between the push and pull phases may reduce the repetitive overuse of anterior shoulder structures—such as the pectoralis major and supraspinatus—which are frequently involved in propulsion-related pathologies [3,4]. Supporting this rationale, Mulroy et al. [26] analyzed shoulder muscle activity in individuals with paraplegia during wheelchair propulsion. They observed distinct differences in electromyographic patterns between the push and recovery phases, with significantly elevated loads on anterior musculature, particularly during push strokes. Such findings suggest that propulsion strategies that distribute muscular workload more evenly across different phases of motion may reduce fatigue and help prevent long-term musculoskeletal injuries. In line with this, our dual-propulsion system is designed to lower peak kinetic loads and balance activation across muscle groups. If confirmed using EMG and kinematic analyses, these effects may contribute to reducing the risk of shoulder overuse injuries among manual wheelchair users.

Usability is another crucial factor influencing the adoption of new propulsion technologies. Cowan et al. [27] demonstrated that heavier wheelchair configurations increase propulsion force requirements, especially in older adults with limited upper body strength. These findings underscore the importance of lightweight and user-centered design in assistive mobility devices.

The current dual-propulsion device was specifically developed to address these issues. Its detachable and lightweight structure is intended to improve portability, while the intuitive push–pull mechanism enhances ease of use, especially for novice users. These characteristics aim to reduce the physical burden of propulsion and make the system more accessible to a broader range of manual wheelchair users.

Beyond usability, this study emphasizes the integration of objective biomechanical performance measures in the evaluation of assistive devices. Following the recommendation by Dittli et al. [13], the protocol includes synchronized motion capture and surface EMG to quantify propulsion mechanics and muscular engagement across propulsion modes.

By adopting a structured biomechanical evaluation framework, the study builds upon existing knowledge in wheelchair propulsion and expands it using a novel dual-propulsion design. This evidence-based engineering and clinical approach strikes a balance between innovation and practical feasibility. If our findings confirm the expected benefits—namely, lower peak shoulder loading and more symmetrical muscle activation—this study may provide meaningful contributions to both assistive technology development and rehabilitation science, while also offering evidence supporting the applicability of dual-propulsion in real-world settings.

## 4. Conclusions

This study protocol proposes a novel detachable dual-propulsion system designed to enhance the biomechanics and usability of manual wheelchairs. By employing synchronized EMG and motion analysis within a within-subjects evaluation framework, the protocol will enable detailed comparisons between forward and backward propulsion. Depending on the distribution of the collected data, appropriate parametric or non-parametric analyses will be applied. The anticipated outcomes may provide foundational insights for future clinical applications and device refinements aimed at reducing injury risk and improving mobility in manual wheelchair users.

## Figures and Tables

**Table 1:** Participant timeline and procedures.

Phase	T0: Pre-Test	T1: Familiarization	T2: Push Trials	T3: Rest	T4: Pull Trials	T5: Post-Test (MVC)
Consent and Demographics	✔️					
Device Instruction		✔️				
Practice (3 min)		✔️				
Kinematic Recording			✔️ (5 × 10 m)		✔️ (5 × 10 m)	
EMG Recording			✔️		✔️	✔️ (3 reps × 3 s)
Rest Interval (≥5 min)				✔️		

## Data Availability

The data will be made available by the authors upon reasonable request.
